# Supplementary figures and images for: SMER28 Attenuates PI3K/mTOR Signaling by Direct Inhibition of PI3K p110 Delta
Source: Cells. 2022 May 16;11(10):1648. doi: 10.3390/cells11101648 (PMC9140127; doi:10.3390/cells11101648)

**Figure S1**

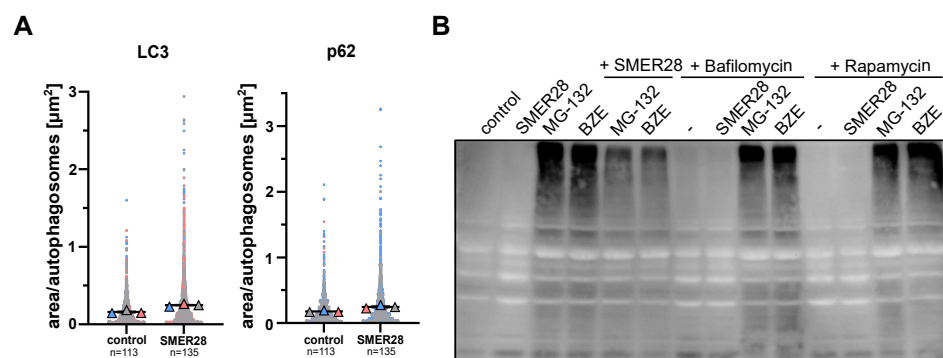

Supplement: Supplementary file 1 [file cells-11-01648-s001.zip › Supplementary Figure S1.pdf]

## Supplementary Figure S2

**A**

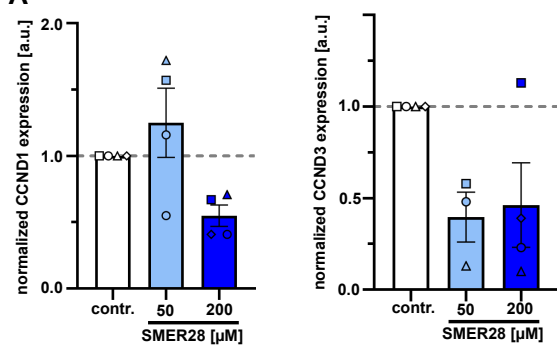

Supplement: Supplementary file 1 [file cells-11-01648-s001.zip › Supplementary Figure S2.pdf]
